# Effectiveness of a Dyadic Buddy App for Smoking Cessation: Randomized Controlled Trial

**DOI:** 10.2196/27162

**Published:** 2021-09-09

**Authors:** Philipp Schwaninger, Corina Berli, Urte Scholz, Janina Lüscher

**Affiliations:** 1 Applied Social and Health Psychology Department of Psychology University of Zurich Zurich Switzerland; 2 University Research Priority Programme “Dynamic of Healthy Aging” Department of Psychology University of Zurich Zurich Switzerland

**Keywords:** mHealth, smartphone app, smoking cessation, buddy, social support, mobile phone

## Abstract

**Background:**

Tobacco smoking is one of the biggest public health threats. Smartphone apps offer new promising opportunities for supporting smoking cessation in real time. This randomized controlled trial investigated the effectiveness of an app that encourages individuals to quit smoking with the help of a social network member (*buddy*) in daily life.

**Objective:**

The objective of this study is to test the effectiveness of the SmokeFree buddy app compared with a control group with self-reported smoking abstinence and carbon monoxide (CO)–verified smoking abstinence as primary outcomes and self-reports of smoked cigarettes per day (CPD) as a secondary outcome.

**Methods:**

A total of 162 adults who smoked participated in this single-blind, two-arm, parallel-group, intensive longitudinal randomized controlled trial. Around a self-set quit date (ie, 7 days before the self-set quit date and 20 days after) and 6 months later, participants of the intervention and control groups reported on daily smoking abstinence and CPD in end-of-day diaries. Daily smoking abstinence was verified via daily exhaled CO assessments. This assessment was administered via an app displaying results of exhaled CO, thus addressing self-monitoring in both groups. In addition, participants in the intervention group used the SmokeFree buddy app, a multicomponent app that facilitates social support from a buddy of choice.

**Results:**

A significant reduction in CPD from baseline to the 6-month follow-up was observed among participants in both groups. Multilevel analyses revealed no significant intervention effect on self-reported and CO-verified daily smoking abstinence at the quit date and 3 weeks later. However, CPD was lower at the quit date and 3 weeks later in the intervention group than in the control group. No significant differences between groups were found for any outcome measures 6 months after the quit date. Overall, low app engagement and low perceived usefulness were observed.

**Conclusions:**

Despite some encouraging short-term findings on the amount of smoking, the SmokeFree buddy app did not have beneficial effects on smoking abstinence over and above the self-monitoring control condition. Future studies should examine whether and what support processes can be effectively stimulated and how app use can be improved to better achieve this goal.

**Trial Registration:**

ISRCTN Registry 11154315; https://www.isrctn.com/ISRCTN11154315

**International Registered Report Identifier (IRRID):**

RR2-10.1186/s12889-019-7723-z

## Introduction

### Background

Smoking kills more than 8 million people each year and remains one of the leading preventable causes of premature death worldwide [1,2]. Cigarette smoking is a major risk factor for life-threatening diseases such as lung cancer, coronary heart disease, and stroke and other noncommunicable diseases [2]. Despite the evidence that quitting smoking is associated with rapid improvements in health and reduced risk for noncommunicable diseases, in 2017, every fourth person aged 15 years and older smoked in Switzerland [3]. The high relapse rates indicate that quitting is difficult [4]. Various socioenvironmental stimuli associated with smoking may lead to high-risk situations for relapse, even long after quitting [5]. Strengthening the capability to manage such high-risk situations (eg, cue-induced cravings) is crucial for smoking cessation interventions [6,7]. The use of mobile health technologies (eg, smartphone apps) offers promising opportunities for smoking cessation interventions in everyday life, with the potential to deliver support in situations when it is most needed [8,9].

### Smartphone Apps to Promote Smoking Cessation

In Switzerland, 92% of adults owned a smartphone in 2018 [10]. Considering this high prevalence of smartphone use, delivering smoking cessation interventions using smartphone apps is a promising approach. With the widespread reach and proximity to the user, interventions are accessible in everyday life [11]. The location and time independence may reduce perceived barriers to treatment (eg, transportation, time, and cost) and make potential engagement convenient [11,12]. Furthermore, apps can provide interactive and tailored intervention features as well as an interactive platform to allow instant sharing of experiences and resources (eg, social support) [11,12]. All these factors contribute to the cost-effectiveness of smoking cessation app interventions [11].

Considering the potential of app interventions, existing apps predominantly provide simple tools (eg, calculators, calendars, trackers, or distractors) [13,14]. The majority of available apps had very little evidence-based content to support quit attempts, such that in 55% of the apps, no behavior change techniques (BCTs) [15] were present [16]. In addition to the content of the smoking cessation app, their effectiveness is less clear. In the context of text messaging interventions for smoking cessation, research has found that such interventions are effective in improving smoking cessation rates and reducing health service costs [17-19]. In contrast, despite the wide availability of smoking cessation apps and the rapid release of new apps [13], only a few studies have tested their effectiveness [17]. For instance, a smartphone decision aid app including behavioral support was compared with a simpler information-only app in a randomized controlled trial (RCT) across different countries [20]. The decision aid app significantly increased continuous smoking abstinence at 1, 3, and 6 months after quitting [20]. A recent review of five studies that tested the effectiveness of smoking cessation apps showed no clear evidence for increasing quit rates [17]. There is a lack of scientific evidence on the effectiveness of smoking cessation apps. We echo the conclusion of the authors that more RCTs with long-term follow-ups (at least 6 months; Russell Standard [21]) are needed to provide evidence on the effectiveness of smoking cessation apps [17]. Therefore, this RCT tests the effectiveness of the SmokeFree buddy app, which was part of the SmokeFree campaign of the Federal Office of Public Health in Switzerland. It is a multicomponent app that, in particular, aims to facilitate social support from a buddy of one’s own social network.

App features that connect individuals who smoke with health care workers, smokers and ex-smokers, or persons from their own social network to foster social support resources are promising [5]. Social support is conceptualized as coping assistance, that is, the supporters’ engagement in coping efforts through the provision of emotional help (eg, demonstration of understanding and valuing) and practical assistance (eg, advice and informational and instrumental support) [22]. Social support may help individuals cope with high-risk situations for smoking (eg, cue-induced cravings) or buffering stress while quitting [9,23]. Research shows that face-to-face individual counseling, group therapy, and telephone counseling that convey social support (eg, practical assistance by identifying high-risk situations or emotional help by encouragement) were found to be effective for smoking cessation [24-26]. Partner or peer support interventions, however, did not show clear effectiveness in smoking cessation [9,27]. These intervention approaches are very diverse, and one explanation is that these partner or peer interventions were not successful in increasing social support in the first place [27]. Furthermore, research shows that support receipt might have null or negative effects on recipients depending on the timing, content, and way how support is provided [28]. More evidence regarding partner or peer support interventions is needed [9,27,29]. Apps provide promising potential to enhance partner or peer support resources and availability during actual experiences in people’s everyday lives [30]. Content analyses of smoking cessation apps, however, have found that apps rarely reference users outside of the app to a quit helpline or provide opportunities to reach out for social support [13]. Only 2.7% (6/225) of rated Android smoking cessation apps had content regarding social support [14]. This RCT tested the SmokeFree buddy app that fosters support resources from a buddy of choice. This app offers theory-based instructions for the buddy on how to support individuals who smoke during a quit attempt. The instructions may positively influence the quality of support, and the accessibility of the app is important for the ideal timing of support exchanges (ie, when it is most needed). In addition, individuals who smoke rely on persons from their own personal social network rather than on unacquainted buddies. This may influence the tailoring of support exchanges because a buddy of choice should know the target person and their needs better than a stranger [31].

### Aims of This Study

Given the research gaps outlined above, this study aims to test the effectiveness of the SmokeFree buddy app in daily life. The SmokeFree buddy app was developed by the Federal Office of Public Health in Switzerland in collaboration with the Institute of Global Health of the University of Geneva, Switzerland. The app is designed to be used simultaneously by an individual who smokes and a personal buddy of choice. In brief, a chat function is the direct communication channel for all text messages and connects the individual who smokes and the personal buddy. The individual who smokes can, for example, indicate the current mood state and intensity and can use notification buttons such as sending an emergency message, communicating craving to smoke, or communicating a lapse. The app then informs the buddy about text messages and notifications and the current mood or craving and provides them with the option of reacting immediately, for example, with a supportive message. The app further provides evidence-informed background information (eg, how to cope with craving) as well as preset supportive messages for the buddy, which can be customized or supplemented with a personal comment. A performance statistic shows the achievements to date (eg, number of smoke-free days), and a knowledge base contains information on smoking and quitting, which is also visible and accessible to the buddy. For more details about the SmokeFree buddy app, please refer to the study protocol [32].

This RCT examined whether the SmokeFree buddy app promotes daily abstinence rates and reduces the number of cigarettes smoked per day (CPD) in adult participants who smoke at a self-set quit date, 3 weeks later (end of intervention), and 6 months later in comparison with a control group (CG) that did not use the SmokeFree buddy app [32]. We hypothesized that adult participants in the intervention group (IG) will show higher daily abstinence rates and lower CPD at the self-set quit date, 3 weeks later (end of intervention), and 6 months later than those in the CG (hypothesis 1). Furthermore, our RCT uses intensive longitudinal data to test the app in participants’ everyday lives, which additionally allows the analysis of the potential time effects of the intervention. Therefore, we additionally assumed a higher increase in daily abstinence rates and a higher decrease in CPD over time in the IG than in the CG (hypothesis 2). The hypotheses were prospectively registered (ISRCTN 11154315). The primary outcome measures were daily self-reported smoking abstinence and daily smoking abstinence using exhaled carbon monoxide (CO) [32]. This RCT assessed smoking abstinence objectively on a daily basis with the iCO Smokerlyzer (Bedfont Scientific Ltd [33]), a CO-monitoring device. The secondary outcome measure was self-reports of smoked CPD [32].

## Methods

### Overview

We conducted a single-blind, two-arm, parallel-group, intensive longitudinal RCT *smoking cessation with smartphone apps* (for a detailed description, please see the study protocol [32]). The trial consisted of a *baseline diary* (3 consecutive days), a background assessment, a *challenge diary* from 7 days before the self-set quit date until 20 days after the self-set quit date (28 consecutive days), and a *follow-up diary* 6 months after the self-set quit date (3 consecutive days). Participants were randomly allocated to the intervention (SmokeFree buddy app) or control condition (no SmokeFree buddy app). This trial was approved by the Ethics Committee of the Faculty of Arts and Social Sciences of the University of Zurich (reference number: 17.12.13) and was prospectively registered (ISRCTN11154315).

### Participants and Procedures

Participants were adults who smoked at least one cigarette per day, intended to quit smoking during the study, and owned a smartphone with access to mobile internet. The exclusion criteria were insufficient knowledge of the German language, working in 24-hour shifts, use of a smoking cessation app, or participation in a professional smoking cessation program. Participants were recruited via advertisements in newspapers, web-based platforms, webpages, and flyers at the university, medical facilities, and local companies from April 2018 to August 2019 in Switzerland. The eligibility criteria were assessed using a web-based screening questionnaire. Participants started with a web-based end-of-day diary questionnaire for 3 consecutive days (*baseline diary*). After completion, participants were invited to the lab for a background assessment. The interviewer conducting the background assessment randomly assigned participants to one of the two groups (IG and CG), according to a computer-generated allocation sequence that was concealed in a set of sealed, numbered envelopes. Participants were blinded to the group assignment (ie, single-blind RCT; for the detailed randomization procedure, refer to the study protocol [32]). At the background assessment, all participants provided written informed consent, completed a questionnaire, and were asked to self-set a quit date (BCT *goal setting of behavior* [15]) within the next 6 weeks. The self-set quit date determined the start of the 28-day diary period (*challenge diary*), starting 7 days before their self-set quit date. Furthermore, all participants received a personal iCO Smokerlyzer and instructions on how to use it with the corresponding app to measure the exhaled CO daily. The first measurement of CO with the iCO Smokerlyzer was conducted at the background assessment. All participants were instructed to fill out an end-of-day diary (daily text message with link to questionnaire) and to measure the exhaled CO daily using the personal iCO Smokerlyzer for 28 consecutive days during the *challenge diary* phase (7 days before the self-set quit date, on the self-set quit date until 20 days after the self-set quit date). Six months after the self-set quit date, participants in the CG and IG were asked to participate in the *follow-up diary* phase, with end-of-day diaries and daily measures of exhaled CO using the personal iCO Smokerlyzer for 3 consecutive days. As an incentive for study participation, all adults who smoke received a personal iCO Smokerlyzer with a value of CHF 60 (US $63). Furthermore, with completion of the 6-month follow-up, participants were given entry into a lottery with a main prize of CHF 200 (US $209) and 40 prizes with a value of CHF 50 (US $52) shopping vouchers. Buddy participants were reimbursed with CHF 50 (US $52) at the completion of the study. On the basis of a power of 0.80 and a two-tailed type 1 error probability of *P*=.05, an a priori power analysis yielded a sample size of 128 to detect a 24% difference in smoking abstinence for the IG compared with the CG, drawing on meta-analyses of mobile phone interventions on short- and long-term smoking abstinence (rate ratios [RRs] between 1.7 and 2.1 [34,35]). Assuming an attrition rate of 25%, 6 months after the quit date resulted in a total sample size of 160 participants (80 participants per group). For more details, please refer to the study protocol [32].

### Intervention

As described above, participants in the CG and IG set a self-set quit date and were instructed to fill out the end-of-day diaries and to measure exhaled CO with the iCO Smokerlyzer during the *challenge diary* phase. The CO Smokerlyzer app displayed participants’ CO results and progress with smoking cessation and thus addressed the BCTs *feedback on behavior* and *self-monitoring of behavior* [15]. Only the IG participants were additionally introduced to the SmokeFree buddy app. Participants in the IG were instructed to identify a personal buddy of choice and to use the SmokeFree buddy app during the *challenge diary* phase (starting 7 days before the self-set quit date). The SmokeFree buddy app is available for free in the App Store and Google Play Store (version 4.0). The app comprises the following BCTs: *social support (emotional), social support (practical), social reward, feedback on behavior, self-monitoring of behavior, others monitoring with awareness, discrepancy between current behavior and goal standard, nonspecific reward,* and *information about health consequences* [15]. The buddy had to be a nonsmoker for at least 6 months, owning a smartphone with access to mobile internet. The exclusion criteria for the buddy were insufficient knowledge of the German language and working in 24-hour shifts. The buddy also provided informed consent and received a background questionnaire with instructions regarding the SmokeFree buddy app. The buddy participants were also instructed to fill out an end-of-day diary and to use the SmokeFree buddy app during the *challenge diary* phase (for 28 consecutive days) to interact with the participant who smoked. No recommendations regarding timing, frequency, and intensity of use were provided to participants who smoked and their buddies.

### Measures

#### Primary Outcome: Daily Smoking Abstinence (Self-reported and CO Verified)

Participants indicated in a daily evening diary questionnaire whether they smoked that day or not by answering the question, “Did you smoke today (including only one puff)?” (coded as 0=yes and 1=no) [36,37]. To objectively assess smoking abstinence, participants were instructed each evening to assess exhaled CO with their personal iCO Smokerlyzer. The iCO Smokerlyzer is a CO-monitoring device that is used with a corresponding Smokerlyzer app [33]. At the end of the end-of-day diary questionnaire, participants were instructed to take a CO measurement with their personal iCO Smokerlyzer and the corresponding app. The Smokerlyzer app displayed the test values in parts per million (ppm) and automatically generated a result report. Participants were instructed to send their result report directly from the app via email to the study team and to report the ppm value in the end-of-day diary. The iCO Smokerlyzer test values were categorized as 1 for abstinent (0-6 ppm) and as 0 for not abstinent (>6 ppm) [33,38]. A recent study points to comparable results between the iCO Smokerlyzer and the more studied piCO^+^ Smokerlyzer, which has been shown to validly distinguish between smokers and nonsmokers [38,39].

#### Secondary Outcome: CPD

If participants answered the smoking abstinence measure with yes (=today smoked), they were asked to report the number of cigarettes they had smoked that day. Otherwise, the daily number of cigarettes smoked was coded as 0 [37].

#### Covariates

The daily use of nicotine replacement products (NRPs) and a variable denoting whether days were weekdays or weekends were included as covariates in the sensitivity analyses. Research shows that using NRPs increases the probability of smoking cessation success [40], and smoking behavior has been shown to differ during weekends [41], which might be related to trigger exposure. NRPs were not provided to participants; however, the SmokeFree buddy app (ie, the knowledge base feature) incorporated encouragement to use NRPs for smoking cessation. Participants indicated in the daily diary whether they used NRPs or not by answering the question “Did you use nicotine replacement products today (eg, nicotine gum, nicotine patch, medications as for example Champix, etc)?” (0=no and 1=yes). Furthermore, with each timestamp of the daily diary, a variable was calculated, whether it was a weekend or not (0=no weekend and 1=weekend).

### Data Analysis

#### Intention to Treat

In line with the outcome criteria recommended for smoking cessation trials (Russell Standard [21]), our final models included all randomized subjects (intention to treat). Participants who were lost to the *challenge* or *follow-up diary* phase were treated as smoking for the analyses, with the primary outcome variables of daily abstinence (0=not abstinent and 1=abstinent).

#### Analysis of the Intervention Effect at the Quit Date and End of the Intervention

To account for the nested structure of repeated measures within individuals, multilevel modeling was used [42]. To examine the intervention effect on the two dichotomous primary outcome measures, we ran logistic regressions using generalized linear mixed models (GLMMs). The continuous outcome measure, CPD, was a count variable that was highly skewed with a large number of zeroes. Therefore, we applied a GLMM for count outcomes using a negative binomial distribution with a logarithmic link function [43]. Owing to the logarithmic link function, regression coefficients are on a log scale and interpreted as RRs. The distance to an RR of 1 is interpreted as the percentage increase (above one) or decrease (below one) in the outcome for a one-unit increase in the predictor [44]. The specified multilevel models use all available diary entries using maximum likelihood estimations [42]. All analyses were conducted using SPSS 26.

To test our research question and corresponding hypotheses, all outcome measures were modeled as a function of *time*, *group* (coded as 0=CG and 1=IG; hypothesis 1), and *group by time* (hypothesis 2). For this purpose, a linear *time* variable for the 28 consecutive days of the *challenge diary* was created (in 1-day units). To test group differences at the quit date and 3 weeks later, the *time* variable was centered accordingly (centered on the quit date: day 8=0 or centered on the last day: day 28=0). An initial graphic display of the data showed a discrete change in the outcomes at the quit date, indicating two qualitatively distinct phases before and after. To model this nonlinear trajectory over time (shift), a dummy-coded variable *quit* was computed (coded as 0=quit day and days after the quit date [days 8-28] and 1=days before the quit date [days 1-7]). To test group differences in this shift, we included an interaction term of the *quit* and the *group* variable (*group by quit*). Finally, to test for differential time effects before and after the quit date, we generated and added a second time variable reflecting an interaction between *quit* and *time* (ie, *quit by time* with the following coding: day 1=−7, day 2=−6, ..., day 7=−1, day 8=0, day 9=0, day 10=0, etc). No differential time effects of the two groups before the quit date emerged; therefore, this interaction was not included in the final model. In all analyses, we routinely included the grand-mean-centered CPD during the *baseline diary* phase as an indicator of nicotine dependence as a covariate. All models specified a maximal random effects structure, which, in the case of nonconvergence, was successively reduced until convergence was achieved [45].

#### Analysis of the Intervention Effects at Follow-up

To investigate the intervention effect 6 months after the quit date, group differences regarding the primary and secondary outcome measures were examined by conducting independent samples two-tailed *t* tests (averaged days 1-3 of the *follow-up diary* phase per person). If variances were unequal between the groups, Welch *t* test was used. We used a Bonferroni-adjusted significance level to account for multiple comparisons.

## Results

### Sample

**Figure 1:**
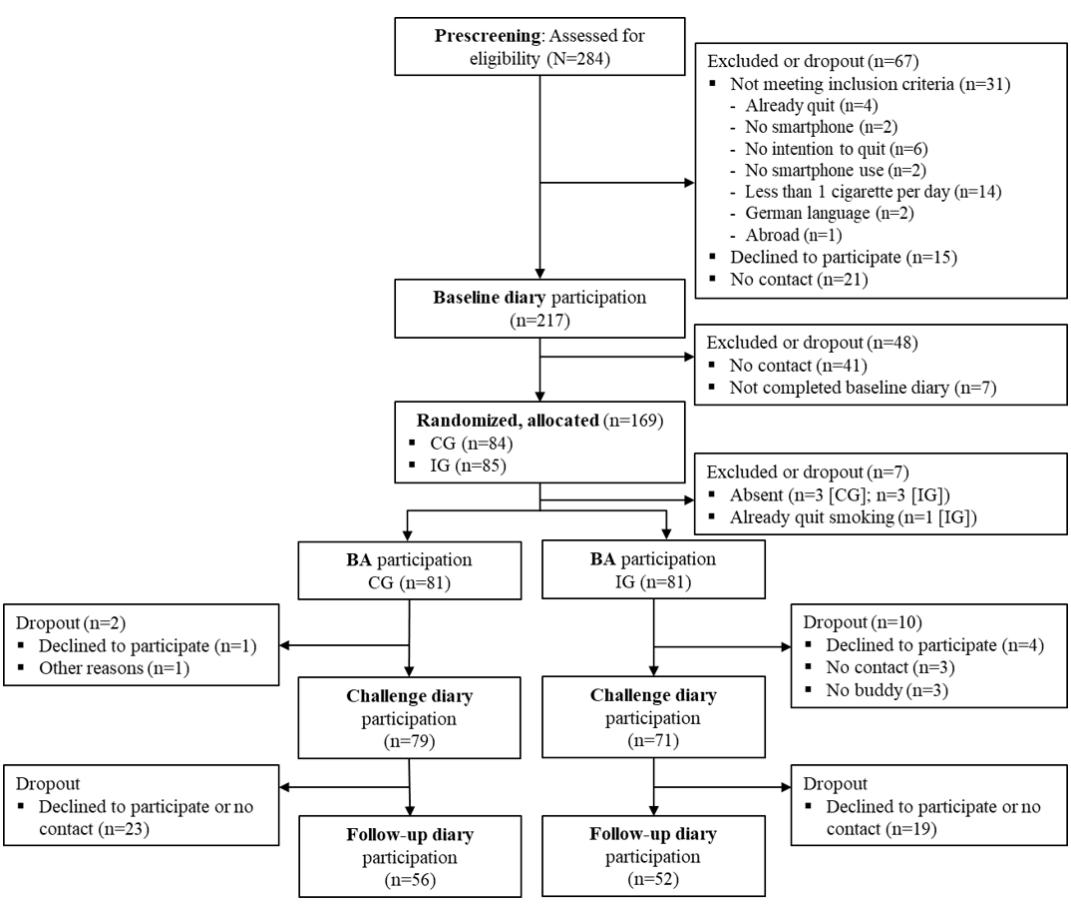
Participant flow. BA: background assessment; CG: control group; IG: intervention group.

A total of 284 participants were screened, and 169 (59.5%) participants who completed the *baseline diary* were randomized to the CG (n=84 in total) and IG (n=85 in total; refer to Figure 1 for the participant flow).

Overall, 162 randomized participants attended the background assessment, 81 in the CG and 81 in the IG. Table 1 displays the background characteristics of the participants in the CG and IG. Participants in the CG and IG did not differ on these variables at the background assessment (Table 1), proving successful randomization. The 162 participants had a mean age of 31.3 (SD 10.9) years and smoked 12.8 CPD (SD 7.1) before study participation. The majority of the participants were male (89/162, 54.9%). There were no significant background differences between groups regarding the intention and the desire to stop smoking reported at the background assessment as well as the self-regulation variables such as self-efficacy, action control, and social support receipt reported during the *baseline diary* (Table 1).

Of the 162 participants, 12 (7.4%) did not start with the *challenge diary*. By treating the dropout as smoking (intention to treat), the analyses of the primary outcomes had the maximum number of available data points (N=162; 3339/4536, 73.61% possible data points). Due to technical problems with the iCO Smokerlyzer, there were additional missing values for the CO-verified abstinence measure. CO measures were completely missing for 8 participants (103 missing data points). Additional missing CO values occurred due to various reasons, such as iCO Smokerlyzer did not work (57 days), iCO Smokerlyzer or smartphone forgotten (36 days), no time (5 days), or other unknown reasons (34 days). Therefore, the analysis of the CO-verified abstinence measure had 235 additional missing data points (n=154; 3104/4536, 68.43% possible data points). Secondary outcome data, CPD, were available for 97% (79/81) of the participants in the CG and 88% (71/81) of the participants in the IG (total n=150; 3003/4536, 66.2% possible data points).

**Table 1:** Baseline characteristics of the target persons in the intervention group and control group (N=162).

Variables			Intervention group (n=81)		Control group (n=81)		Intervention group vs control group^a^		*P* value
**Gender, n (%)**							0.2 (1)^b^		.64
	Female	35 (43)		38 (47)					
	Male	46 (57)		43 (53)					
Unmarried, n (%)			65 (80)		64 (79)		3.7 (2)^b^		.16
Higher education, n (%)			32 (40)		38 (47)		2.2 (3)^b^		.53
Employed, n (%)			35 (43)		41 (51)		3.1 (4)^b^		.54
Age (years), mean (SD)			31.32 (11.50)		31.32 (10.27)		0.00 (160)^c^		.99
Daily number of cigarettes (background assessment), mean (SD)			13.07 (7.33)		12.44 (6.83)		−0.57 (160)^c^		.57
Nicotine dependence, mean (SD)			3.33 (2.08)		3.56 (2.17)		0.67 (160)^c^		.51
Exhaled carbon monoxide (ppm^d^), mean (SD)			9.81 (6.34)		10.65 (9.05)		0.67 (139)^c^		.50
Intention to stop smoking, mean (SD)			5.40 (0.71)		5.33 (0.77)		−0.58 (160)^c^		.56
Desire to stop smoking, mean (SD)			5.00 (0.70)		4.95 (0.67)		−0.46 (160)^c^		.65
Baseline self-efficacy^e^, mean (SD)			2.83 (1.34)		2.72 (1.24)		−0.55 (160)^c^		.59
Baseline action control, mean (SD)			2.48 (1.06)		2.75 (1.11)		1.54 (160)^c^		.13
Baseline social support receipt, mean (SD)			1.22 (1.87)		1.56 (1.85)		1.18 (160)^c^		.24

^a^Groups were compared for baseline characteristics using independent *t* tests for continuous data and chi-square tests for categorical data.

^b^Chi-square (*df*) values for categorical data.

^c^*t* test (*df*) values for continuous data.

^d^ppm: parts per million.

^e^Baseline variables were assessed during the *baseline diary* phase.

In total, 108 participants completed the *follow-up diary* phase (Figure 1). There were no significant differences in terms of background characteristics between participants who dropped out of the study and participants with follow-up data (Table S1 in Multimedia Appendix 1). By treating the dropout as smoking (intention to treat), the analyses regarding the primary outcomes included the full sample (N=162). Due to missing or not working iCO Smokerlyzer devices, the CO-verified abstinence measure was available for 86.4% (140/162) participants. Secondary outcome data were available for 66.7% (108/162) participants.

### Descriptive Results

**Figure 2:**
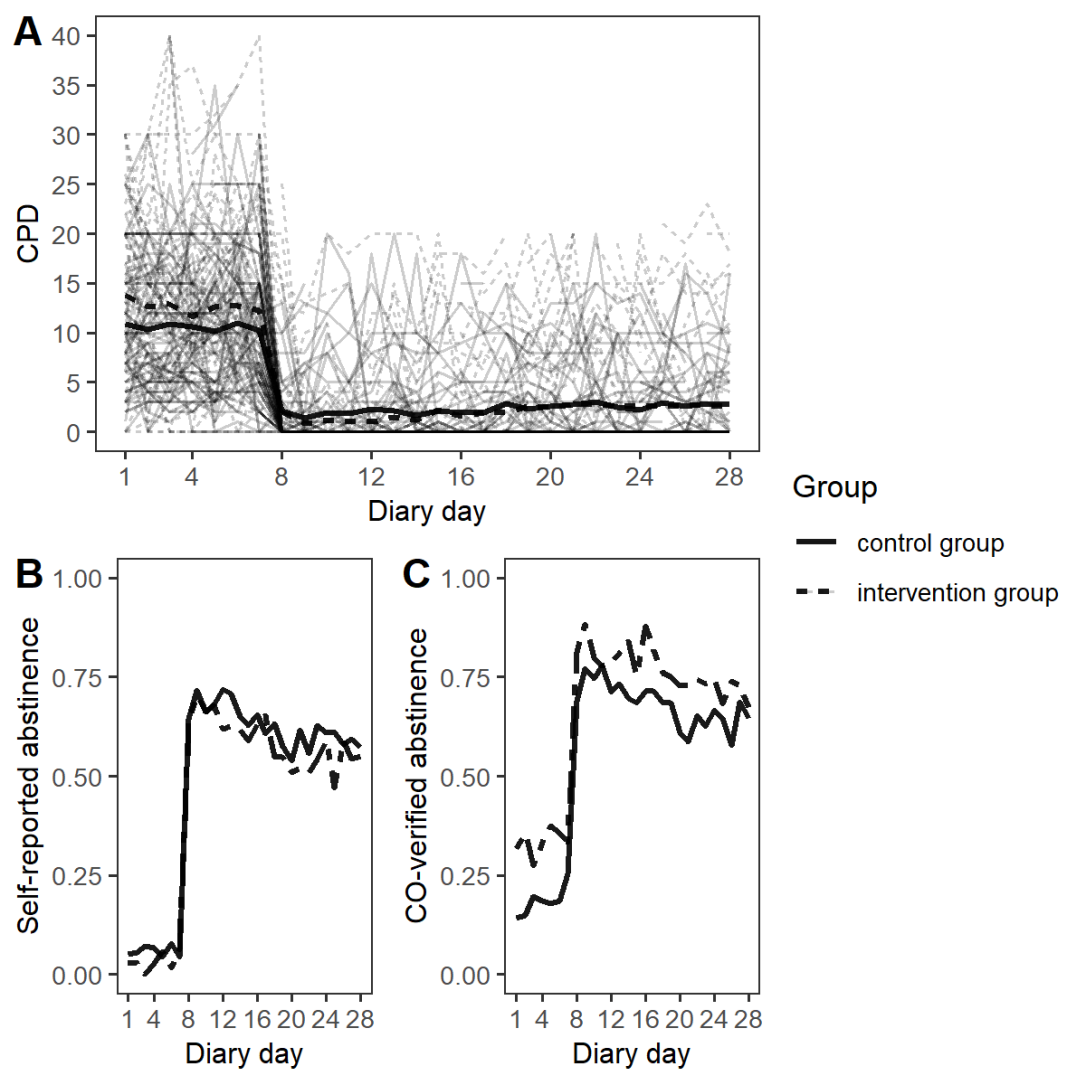
Means of cigarettes per day (A), self-reported abstinence (B) and carbon monoxide–verified abstinence (C) over 28 days of the challenge diary phase by groups. CPD: cigarettes per day; CO: carbon monoxide.

Table 2 shows the average CPD during the *baseline diary* phase and *challenge diary* phase and the 3 weeks and 6 months continuous abstinence rates by groups. The IG had a higher 3 weeks and 6 months continuous abstinence rate than the CG; however, these differences did not reach significance. For the CG, dependent *t* tests indicated a significant decrease in CPD from the baseline diary to the challenge diary *(postquit; t*_72_=11.29; *P*<.001; *d*=2.66) and from the baseline diary to the follow-up diary (t_55_=6.14; *P*<.001; *d*=1.66). The IG also showed a significant decrease in CPD from the baseline diary to the challenge diary *(postquit; t*_70_=13.25; *P*<.001; *d*=3.17) and from the baseline diary to the follow-up diary (t_51_=6.51; *P*<.001; *d*=1.82). Figure 2 displays the means of the three outcome measures over the 28 days of the *challenge diary* by groups.

**Table 2:** Average cigarettes per day during the three diary phases and 3 weeks and 6 months continuous abstinence rates for the intervention group and control group (N=162).

Variables	Intervention group (n=81)	Control group (n=81)	Intervention group vs control group^a^	*P* value
CPD^b^ baseline diary (N=162), mean (SD)	12.41 (7.50)	11.48 (7.08)	−0.81 (160)^c^	.42
CPD challenge diary prequit (n=149), mean (SD)	12.31 (7.29)	10.48 (6.15)	−1.67 (147)^c^	.10
CPD challenge diary postquit (n=144), mean (SD)	1.99 (4.03)	2.51 (3.14)	0.86 (142)^c^	.39
CPD follow-up diary (n=108), mean (SD)	6.13 (8.17)	4.99 (5.74)	−0.84 (91)^c^	.40
3 weeks continuous abstinence (ITT^d^) (N=162), n (%)	25 (30.9)	19 (23.5)	1.1 (1)^e^	.19
6 months continuous abstinence^f^ (ITT) (N=162), n (%)	18 (22.2)	11 (13.6)	2.1 (1)^e^	.11

^a^Groups were compared for number of cigarettes per day and continuous abstinence rates using independent *t* tests for continuous data and chi-square tests for categorical data.

^b^CPD: cigarettes per day; the available data for the variable cigarettes per day varied due to dropout.

^c^*t* test (*df*) values.

^d^ITT: intention to treat; lost participants were treated as smoking (full sample).

^e^Chi-square (*df*) values.

^f^Six months continuous abstinence is defined as ≤5 cigarettes since quit date and no smoking during the follow-up diary phase (carbon monoxide verified).

### Intervention Effects at the Quit Date and End of Intervention

The results of the three GLMMs are presented in Tables 3 and 4. In terms of daily self-reported smoking abstinence (GLMM 1), the intercept describes the probability of smoking abstinence for the CG at the quit date (77.4%, 95% CI 59.4%-88.9%). No significant *group* effect emerged: the IG (85.1%, 95% CI 42.4%-97.8%) did not report higher smoking abstinence than the CG. No significant *time* trend and no significant *group-by-time* effect were observed. This indicates that the daily self-reported abstinence was stable for both groups from the quit date on (disconfirming hypothesis 2), and the nonsignificant group difference on the quit date remained until the end of the intervention (disconfirming hypothesis 1; probability of abstinence on day 28: CG: 50.2%, 95% CI 17.2%-83.1% and IG: 74.2%, 95% CI 6.2%-99.2%). These results are not in support of our hypotheses. However, for both groups, a significant increase in the probability of daily self-reported abstinence from the phase before the quit date to the phase after the quit date occurred. Before the quit date, no difference was observed in the probability of daily self-reported abstinence between the CG (0.47%, 95% CI 0.05%-4.3%) and the IG (0.66%, 95% CI 0.1%-27.5%; to test group differences before the quit date, we recentered the time variable accordingly [centered on the first day: day 1=0]). The level-2 random effects indicated considerable variation between participants in the intercepts and trajectories over time (Table S2 in Multimedia Appendix 1).

**Table 3:** Fixed effects of generalized linear mixed models testing the effect of the intervention group versus the control group on daily self-reported abstinence and carbon monoxide abstinence (generalized linear mixed models 1 and 2).

Fixed effects	GLMM^a^ 1^b^ (daily self-reported abstinence) (N=162)				GLMM 2^c^ (daily carbon monoxide abstinence) (n=154)		
	b^d^ (SE)	*P* value	OR^e^ (95% CI)	b (SE)		*P* value	OR (95% CI)
Intercept^f^	1.23 (0.43)	.005	3.43 (1.47-8.01)	3.37 (0.50)		<.001	29.21 (10.88-78.46)
CPD^g^ (baseline diary)^h^	−0.10 (0.03)	.002	0.91 (0.85-0.97)	−0.14 (0.03)		<.001	0.87 (0.82-0.93)
Group (0=control group; 1=intervention group)	0.51 (0.61)	.40	1.67 (0.50-5.55)	−1.01 (0.69)		.14	0.37 (0.10-1.40)
Time^i^	−0.06 (0.04)	.13	0.94 (0.87-1.02)	−0.06 (0.04)		.08	0.94 (0.88-1.01)
Group×time	0.03 (0.06)	.63	1.03 (0.92-1.15)	0.03 (0.05)		.48	1.03 (0.94-1.14)
Quit (0=after quit date; 1=before quit date)	−6.60 (0.72)	<.001	0.001 (0.00-0.01)	−4.36 (0.63)		<.001	0.01 (0.00-0.04)
Group×quit	0.35 (0.91)	.70	1.42 (0.24-8.42)	−0.08 (0.80)		.92	0.92 (0.19-4.43)
Quit×time	−0.003 (0.08)	.97	0.98 (0.86-1.16)	0.15 (0.08)		.06	1.16 (0.997-1.34)

^a^GLMM: generalized linear mixed model. Random effects are reported in Table S2 in Multimedia Appendix 1.

^b^Generalized linear mixed model 1 (logistic regression): N=162 persons with a maximum of 28 days; n=3339 out of 4536 possible diary entries.

^c^Generalized linear mixed model 2 (logistic regression): n=154 persons with a maximum of 28 days; n=3104 out of 4536 possible diary entries.

^d^b: unstandardized regression coefficient.

^e^OR: odds ratio.

^f^Intercept indicates the level of the outcome for the control group at quit date (day 8).

^g^CPD: cigarettes per day.

^h^The grand-mean-centered cigarettes per day during the *baseline diary* phase as covariate.

^i^Linear time trend centered on the quit date (day 8=0).

**Table 4:** Fixed effects of generalized linear mixed model testing the effect of the intervention group versus the control group on smoked cigarettes per day (generalized linear mixed model 3).

Fixed effects	GLMM^a^ 3^b^: CPD^c^ (n=150)		
	b^d^ (SE)	*P* value	Rate ratio (95% CI)
Intercept^e^	−0.12 (0.16)	.44	0.88 (0.65-1.21)
CPD (baseline diary)^f^	0.09 (0.01)	<.001	1.09 (1.06-1.12)
Group (0=control group; 1=intervention group)	−0.71 (0.23)	.002	0.49 (0.31-0.78)
Time^g^	0.03 (0.01)	.01	1.03 (1.01-1.05)
Group×time	−0.001 (0.01)	.95	0.99 (0.97-1.03)
Quit (0=after quit date; 1=before quit date)	2.52 (0.17)	<.001	12.40 (8.89-17.30)
Group×quit	0.64 (0.23)	.006	1.89 (1.20-2.96)
Quit×time	−0.04 (0.03)	.13	0.96 (0.91-1.01)

^a^GLMM: generalized linear mixed model. Random effects are reported in Table S3 in Multimedia Appendix 1.

^b^Generalized linear mixed model 3 (negative binomial): n=150 persons with a maximum of 28 days; n=3003 out of 4536 possible diary entries.

^c^CPD: cigarettes per day.

^d^b: unstandardized regression coefficient.

^e^Intercept indicates level of the outcome for the control group at quit date (day 8).

^f^The grand-mean-centered cigarettes per day (cigarettes per day) during the *baseline diary* phase as covariate.

^g^Linear time trend centered on the quit date (day 8=0).

The GLMM 2 results with daily CO abstinence as an outcome confirmed the result pattern stated above. The intercept as the probability of CO abstinence for the CG at the quit date was 96.7% (95% CI 91.7%-98.7%). No significant *group* effect emerged: the IG (92.4%, 95% CI 50.1%-99.1%) did not report higher CO abstinence. No significant *time* trend from the quit date on and no significant *group-by-time* effect were observed. This indicates that the daily CO abstinence was stable for both groups from the quit date on (disconfirming hypothesis 2), and the nonsignificant group difference on the quit date remained until the end of the intervention (disconfirming hypothesis 1; probability of CO abstinence on day 28: CG: 89.6%, 95% CI 69.2%-97% and IG: 87.6%, 95% CI 20.7%-99.3%). These results are not in support of our hypotheses. However, a significant increase in the probability of daily CO abstinence from the phase before the quit date to the phase after the quit date occurred for both groups. No significant group differences were observed in the probability of daily CO abstinence before the quit date between the CG (27.2%, 95% CI 3.9%-77.4%) and the IG (25.6%, 95% CI 0.2%-93.8%). The level-2 random effects indicated considerable variation between participants in the intercepts and trajectories over time (Table S2 in Multimedia Appendix 1).

In terms of CPD as an outcome (GLMM 3), the intercept describes the estimated CPD on the quit date in the CG. The average number of cigarettes smoked at the quit date in the CG was low, with about 1 cigarette (0.88) compared with about 12 CPD before the quit date. A significant difference emerged between the IG and CG (b=−0.71, SE 0.23; RR 0.49; *P*=.002). This result indicates that participants in the IG smoked approximately half of the number of cigarettes at the quit date compared with participants in the CG. A significant positive *time* trend (*time*; days 8-28), but no significant *group-by-time* effect emerged. This indicates that for both groups, CPD increased across days from the quit date on. The significant difference between groups remained until the end of intervention, with the CG reporting smoking on average 1.5 cigarettes and the IG on average half the amount (b=−0.73, SE 0.25; RR 0.48; *P*=.003). These results are in support of our hypothesis 1 but not in support of our hypothesis 2, assuming different time trends across groups. A significant decrease in CPD from the phase before the quit date to the phase after the quit date occurred. This nonlinear change in CPD was significantly greater for the IG. The level-2 random effects indicated considerable variation among the participants in the intercepts (Table S3 in Multimedia Appendix 1).

### Sensitivity Analyses

We conducted sensitivity analyses of the intervention effect at the quit date and the end of the intervention to test whether results differed when controlling for the use of NRPs and a variable denoting whether days were weekdays or weekends (see the corresponding results in Tables S4 and S5 in Multimedia Appendix 1). Due to the additional covariates, we could only include participants with *challenge diary* entries (n=150). The result pattern of the models with daily CO abstinence (GLMM A2; Table S4 in Multimedia Appendix 1) and CPD (GLMM A3; Table S5 in Multimedia Appendix 1) as outcome remained the same. In the model with daily self-reported smoking abstinence (GLMM A1; Table S4 in Multimedia Appendix 1), an intervention effect emerged, such that the probability of self-reported abstinence on the quit date was significantly higher for the IG (92.6%, 95% CI 67.2%-98.7%) than for the CG (76.6%, 95% CI 61%-87.2%). This significant difference between groups remained until the end of the intervention (no difference in time trend between groups). The additional covariates were not the reason for the significant group effect on self-reported smoking abstinence, as the effect remained when running a model without the additional covariates (n=150; Table S6 in Multimedia Appendix 1; GLMM A4 and A5). These analyses excluded the dropout participants, that is, the 12 participants without *challenge diary* data (no intention to treat), and therefore estimated group differences less conservatively.

### Intervention Effects at Follow-up

At the 6 months follow-up, regarding the outcome daily self-reported abstinence, we analyzed the full sample (intention to treat) and found no difference between the CG (mean 0.26, SD 0.42; n=81) and IG (mean 0.32, SD 0.46; n=81; t_160_=−0.93; *P*=.36; *d*=−0.15). We found the same result pattern for the daily CO abstinence. No group differences emerged between the CG (mean 0.36, SD 0.47; n=67) and IG (mean 0.40, SD 0.48; n=73; t_138_=−0.48; *P*=.63; *d*=−0.08). The same result patterns were found in the sensitivity analyses when excluding dropouts from the analysis (no intention to treat). For daily self-reported abstinence, no difference emerged between the CG (mean 0.37, SD 0.46; n=56) and IG (mean 0.50, SD 0.49; n=52; t_106_=−1.40; *P*=.16; *d*=−0.27). In addition*,* for daily CO abstinence, no difference emerged between the CG (mean 0.55, SD 0.49; n=44) and IG (mean 0.67, SD 0.46; n=44; t_86_=−1.13; *P*=.26; *d*=−0.24). On average, participants in the CG smoked fewer CPD during the *follow-up diary* phase than participants in the IG (Table 2). This difference was not significant (t_91_=−0.84; *P*=.40; *d*=−0.18).

### Intervention Fidelity

Of the 81 participants in the IG, 10 did not start the *challenge diary* (Figure 1). Of the remaining 71 participants, 1 did not find a buddy but started the *challenge diary* and used the SmokeFree buddy app alone. Excluding this participant did not change the result pattern. To measure app engagement, participants in the IG were asked if they used the SmokeFree buddy app on a daily basis. In addition, the daily perceived usefulness of the app was assessed. Furthermore, we had access to the Flurry Analytics account of the app developer. Flurry Analytics is a platform for tracking and analyzing users’ app engagement (app sessions). Of 47 participants we obtained informed consent to analyze objective data of their SmokeFree buddy app use (Flurry Analytic). Overall, we observed low app engagement and low reported perceived usefulness (Table S5 in Multimedia Appendix 1). A total of 3 of the 71 participants indicated that they had never used the app through the *challenge diary* phase (no objective use data were available). Excluding these 3 participants did not change the result pattern.

## Discussion

### Principal Findings

The aim of this study is to examine the effectiveness of the SmokeFree buddy app, using an RCT with intensive longitudinal data and daily objective measures of smoking abstinence via exhaled CO in participants’ daily lives. The SmokeFree buddy app group was compared with a CG that did not use the SmokeFree buddy app. Both groups set a quit date, filled in daily diaries, and measured exhaled CO with an app targeting self-monitoring and feedback on behavior. First, we hypothesized that the IG will show higher daily abstinence and lower CPD at the quit date, 3 weeks later, and 6 months later than the CG (hypothesis 1). Second, we hypothesized a higher increase or decrease in the outcome measures over time from the quit date to 3 weeks later in the IG than in the CG (hypothesis 2).

Both groups significantly reduced their CPD from the baseline diary phase to the follow-up diary phase. The observed 6 months continuous abstinence rates (CO verified; Table 2) in the IG (18/81, 22%) and CG (11/81, 14%) were promising, as other recent RCTs of smoking cessation apps show continuous abstinence rates (6 months) from 7.8% [46], 10.2% [20] to 16.1% [47]. The CG was found to comparably reduce smoking and achieve similar abstinence rates as the IG. Therefore, our findings do not confirm hypothesis 1 with regard to daily abstinence rates. Regarding the secondary outcome, we found a significantly higher decrease from before to after the quit date in CPD for the IG and significantly lower CPD at the quit date and 3 weeks later, but not 6 months later, compared with the CG. Disconfirming hypothesis 2, we did not find differences in the increase or decrease in the outcome measures over time between the IG and CG. In both groups, the abstinence measures were low prequit, instantaneously shifted to a high level at the quit date, and remained stable during the 3 weeks postquit. In both groups, CPD was high prequit, instantaneously shifted to a low level at the quit date, and then slightly increased during the 3 weeks postquit (Figure 2). At the 6-month follow-up, CPD levels were approximately half of the CPD levels prequit (Table 2).

To our knowledge, this is the first RCT of an app for smoking cessation that focuses particularly on facilitating social support from a personally chosen buddy. Enhancing social support resources should theoretically help individuals to quit smoking [5,9]. There is evidence from intensive longitudinal studies that higher daily support receipt from one’s partner was related to less daily smoking, and effects were more pronounced after a self-set quit date when support is theoretically most needed [23,37]. Previous intervention studies regarding partner or peer social support to improve smoking cessation, however, could not clearly demonstrate the effectiveness of smoking cessation compared with a CG [9,27]. An explanation is that these partner or peer support interventions were not capable of increasing social support in the first place [27]. Our findings match the results of these social support interventions, with no consistent evidence of its effectiveness [27]. The higher decrease and lower levels of CPD at the quit date and 3 weeks later might be explained by the presence of a buddy of choice which increased support and self-regulatory efforts (eg, self-monitoring and self-efficacy) to cope with single smoking events in the short term. However, the analysis of the secondary outcomes did not include all randomized participants. Excluding the 12 participants who did not start the challenge diary phase led to significant intervention effects on self-reported daily smoking abstinence at the quit date and the end of the intervention (Table S6 in Multimedia Appendix 1).

An explanation for the null effects of the intervention could be that the SmokeFree buddy app was compared with a CG that reported on their smoking and assessed CO on a daily basis, which is a strong self-monitoring intervention itself. It might be that the effect of the SmokeFree buddy app over and above the self-monitoring component was smaller than assumed. Another potential explanation for our inconsistent findings might be the low observed app engagement (Table S7 in Multimedia Appendix 1). At the end of the intervention, participants reported their reasons for potentially low app engagement. One reason often mentioned was the preference for established text messaging channels (WhatsApp and SMS text messages), phone calls, or face-to-face communication, which may have made the additional app medium redundant. Several participants also stated that they did not experience any benefits from using the app and did not feel the need to use the app more often. This is in line with reports of the low perceived usefulness of the app (Table S7 in Multimedia Appendix 1) and may have attenuated the evidence-informed support provision of buddy participants. Moreover, some participants also indicated that they generally use few apps, forgot to use the app, or stopped using the app due to relapse. Furthermore, other social exchange processes may have co-occurred, which may have been perceived as aversive or unwanted (eg, social negativity [48]) and so have no beneficial effects on smoking cessation.

Research has shown a general lack of engagement with digital interventions [49]. Low engagement with app content may limit the potential intervention effects [50]. An explanation for the low app engagement and inconsistent findings may be that the main content of the SmokeFree buddy app to enhance social support resources may not be attractive or fruitful for everyone, especially for individuals who do not rely on social relationships during health behavior change (eg, smoking cessation as *solo struggle*) [31,51]. In line with this, the intensive longitudinal data of this study indicate individual heterogeneity regarding the outcome measures at the quit date and in their time trajectories. Future studies should investigate the effects of the SmokeFree buddy app on hypothesized mechanisms to gain more insight into whether and what processes have been stimulated [32]. More research on the engagement with digital interventions and on factors most related to intervention effectiveness is needed (refer to the conceptual framework of Perski et al [50]). For instance, reminders have been found to be a simple strategy to increase user engagement with a digital smoking cessation intervention, such as tailored text messaging or email prompts [52,53]. In addition, human guidance (eg, by the study coordinator or health care worker) may also improve engagement with digital behavior change interventions [54] because human monitoring may lead to a certain accountability to one’s actions or inactions (eg, supportive accountability) [55]. Finally, in the context of social support interventions, we have to deepen our understanding of how, for whom, and under what conditions social support beneficially influences smoking behavior [31].

### Strengths and Limitations

This study had several strengths. First, the study provides the first experimental evidence of the SmokeFree buddy app, an app with a new approach of buddy support in everyday life compared with unacquainted buddies in other intervention studies. Second, we verified our primary outcome measure of self-reported daily smoking abstinence with daily assessments of exhaled CO with the iCO Smokerlyzer. Third, the intensive longitudinal data are a major strength of this study. The daily assessment gave us a more precise display of smoking behavior and verification of continuous abstinence after a quit date compared with macrotime follow-ups only [56]. Furthermore, the intensive longitudinal study design allowed us to investigate the temporal developments of intervention effects and assess the heterogeneity of trajectories between individuals. We assessed self-reported app engagement during the study and measured it in real time. The daily diary design with daily CO measures increases the ecological validity of our data and is the first of its kind.

This RCT also had some limitations that need to be acknowledged. First, this RCT investigated a multicomponent app and thus is not able to disentangle the effects of individual intervention components. Future app intervention development should be guided by the multiphase optimization strategy [57] to identify effective social support components for smoking cessation first before running an RCT that evaluates a multicomponent intervention package [58,59]. Second, our data indicated low app engagement, which may have limited potential intervention effects.

### Conclusions and Implications

Although the abstinence rates of this study are comparable with those of other studies of smoking cessation apps [17] and both conditions (IG and CG) led to significantly decreased CPD, we found that the SmokeFree buddy app was not superior to a CG with primarily self-monitoring components. The app provides evidence for a greater short-term reduction of CPD. There is a need for more research on the effectiveness of and the engagement with smoking cessation apps, considering the wide availability and the rapid release of new smoking cessation apps. App components aimed at enhancing beneficial social interactions with health care providers, partners, or peers to support smoking cessation require further investigation [60]. This study contributes to the scientific evidence on the effectiveness of smoking cessation apps in everyday life.

## Appendix

Multimedia Appendix 1 Supplementary tables regarding the comparison of baseline characteristics between drop-out participants and participants with follow-up data (Table S1); random effects results of the generalized linear models 1-3 (Tables S2 and S3); additional generalized linear models testing effects of the intervention group versus the control group on all three outcomes with additional covariates (Tables S4 and S5); additional generalized linear models without the drop-out participants coded smoking (Table S6); and self-reported daily app use, daily objective app use, and daily reported perceived usefulness of the SmokeFree buddy app (Table S7).

Multimedia Appendix 2 CONSORT-eHEALTH checklist (V 1.6.1).

## Abbreviations

BCT: behavior change technique
CG: control group
CO: carbon monoxide
CPD: cigarettes per day
GLMM: generalized linear mixed model
IG: intervention group
NRP: nicotine replacement product
ppm: parts per million
RCT: randomized controlled trial
RR: rate ratio
