# Supplementary material for: Effectiveness of a Dyadic Buddy App for Smoking Cessation: Randomized Controlled Trial
Source: J Med Internet Res. 2021 Sep 9;23(9):e27162. doi: 10.2196/27162 (PMC8461528; doi:10.2196/27162)
Supplement: Multimedia Appendix 1 [file jmir_v23i9e27162_app1.docx]

**Multimedia Appendices**

**Table S1.** Baseline characteristics of the drop-out participants and the participants with

follow-up data.^a^

| Variables | Drop-out participants (1),  (*n* = 54) | Participants with follow-up data (2), (*n* = 108) | 1 versus 2, $\text{χ}$*^2^* (df),  *t* (df) | *P value* |
| --- | --- | --- | --- | --- |
| Gender, n (%) |  |  | 0.3 (1) | .58 |
| Female | 26 (48) | 47 (44) |  |  |
| Male | 28 (52) | 61 (57) |  |  |
| Unmarried, n (%) | 43 (80) | 86 (80) | 0.3 (2) | .88 |
| Higher education, n (%) | 26 (48) | 44 (41) | 1.7 (3) | .65 |
| Employed, n (%) | 24 (44) | 52 (48) | 4.3 (4) | .36 |
|  |  |  |  |  |
| Age (years), mean (SD) | 30.74 (10.56) | 31.61 (11.05) | −0.48 (160) | .63 |
| Daily number of cigarettes (BA^b^), mean (SD) | 13.02 (7.25) | 12.63 (7.01) | 0.33 (160) | .74 |
| Nicotine dependence, mean (SD) | 3.67 (2.11) | 3.33 (2.13) | 0.94 (160) | .35 |
| Exhaled CO^c^ (ppm^d^), mean (SD) | 11.28 (9.30) | 9.69 (6.87) | 1.22 (158) | .22 |
| Intention to stop smoking,  mean (SD) | 5.33 (0.71) | 5.38 (0.76) | −0.34 (160) | .74 |
| Desire to stop smoking, mean (SD) | 4.99 (0.57) | 4.97 (0.73) | 0.16 (160) | .87 |
| Baseline self-efficacy^e^, mean (SD) | 2.91 (1.34) | 2.71 (1.27) | 0.92 (160) | .36 |
| Baseline action control, mean (SD) | 2.51 (1.19) | 2.67 (1.03) | −0.85 (160) | .40 |
| Baseline social support receipt, mean (SD) | 1.43 (2.04) | 1.38 (1.78) | 0.16 (160) | .87 |

^a^Groups were compared for baseline characteristics using chi-square tests for categorical data (upper section of the table) and independent *t* tests for continuous data (bottom section of the table).

^b^BA: background assessment.

^c^CO: carbon monoxide.

^d^ppm: parts per million.

^e^Baseline variables were assessed during the *baseline diary* phase.

**Table S2.** Random effects of generalized linear mixed models testing the effect of the intervention group versus the control group on daily self-reported abstinence and carbon monoxide abstinence (generalized linear mixed models 1 and 2).

| Random effects (variance) | | GLMM^a^ 1^b^: daily self-reported abstinence (N=162) | | GLMM 2^c^: daily carbon monoxide abstinence (n=154) | |
| --- | --- | --- | --- | --- | --- |
|  | | Estimate (SE; 95% CI) | *P* value | Estimate (SE; 95% CI) | *P* value |
|  | |  |  |  |  |
| **Level 2 (interindividual)**^d^ | | | | | |
|  | Intercept | 11.50 (1.93; 8.27-15.97) | <.001 | 12.40 (2.25; 8.68-17.70) | <.001 |
|  | Time | 0.08 (0.02; 0.05-0.11) | <.001 | 0.04 (0.01; 0.03-0.06) | <.001 |
|  | Quit | 17.74 (3.44; 12.13-25.95) | <.001 | 18.26 (3.72; 12.3-27.2) | <.001 |
|  | Quit×time | — | — | 0.34 (0.08; 0.21-0.55) | <.001 |
| **Level 1 (intraindividual)**^e^ | | | | | |
|  | Residual | 0.33 (0.01; 0.32-0.35) | <.001 | 0.37 (0.01; 0.35-0.40) | <.001 |
|  | Autocorrelation | 0.10 (0.02; 0.05-0.14) | <.001 | 0.06 (0.02; 0.02-0.11) | .007 |

^a^GLMM: generalized linear mixed model. Fixed effects are reported in Table 3 of the manuscript.

^b^Generalized linear mixed model 1 (logistic regression): N=162 persons with a maximum of 28 days; n=3339 out of 4536 possible diary entries.

^c^Generalized linear mixed model 2 (logistic regression): n=154 persons with a maximum of 28 days; n=3104 out of 4536 possible diary entries.

^d^Due to nonconvergence, no random effects for *group×quit* in all models and for *quit×time* in generalized linear mixed model 1 could be estimated.

^e^Level-1 random effects indicated that there was considerable variation within individuals (ie, residuals from the level-1 fitted values) and significant autocorrelation in the level-1 residuals (ie, that adjacent days were more similar than days further apart).

**Table S3.** Random effects of generalized linear mixed model testing the effect of the intervention group versus the control group on smoked cigarettes per day (generalized linear mixed model 3).

| Random effects (variance) | | GLMM^a^ 3^b^: CPD^c^ (n=150) | |
| --- | --- | --- | --- |
|  | | Estimate (SE; 95% CI) | *P* value |
| **Level 2 (interindividual)**^d^ | | | |
|  | Intercept | 1.00 (0.14; 0.76-1.32)^d^ | <.001 |
|  | Time | — | — |
|  | Quit | — | — |
|  | Quit*Time | — | — |
| **Level 1 (intraindividual)**^e^ | | | |
|  | Residual | 0.77 (0.02; 0.72-0.82) | <.001 |
|  | Autocorrelation | 0.34 (0.02; 0.30-0.38) | <.001 |

^a^GLMM: generalized linear mixed model. Fixed effects are reported in Table 4 of the manuscript.

^b^Generalized linear mixed model 3 (negative binomial): n=150 persons with a maximum of 28 days; n=3003 out of 4536 possible diary entries.

^c^CPD: cigarettes per day.

^d^Due to nonconvergence, only a random intercept in generalized linear mixed model 3 could be estimated.

^e^Level-1 random effects indicated that there was considerable variation within individuals (ie, residuals from the level-1 fitted values) and significant autocorrelation in the level-1 residuals (ie, that adjacent days were more similar than days further apart).

**Table S4.** Generalized linear mixed models testing effects of the intervention group versus the control group on daily self-reported abstinence and carbon monoxide abstinence (generalized linear mixed models A1 and A2) with covariates daily nicotine replacement products and weekend.

|  | | GLMM^a^ A1^b^: daily self-reported abstinence (n=150) | | | | GLMM A2^c^: daily carbon monoxide abstinence (n=142) | | | |
| --- | --- | --- | --- | --- | --- | --- | --- | --- | --- |
|  | | b^d^ (SE) | *P* value | OR^e^ | 95% CI | b (SE) | *P* value | OR | 95% CI |
| **Fixed effects** | | | | | | | | | |
| Intercept^f^ | | 1.18 (0.38) | .002 | 3.27 | 1.57-6.81 | 3.05 (0.39) | <.001 | 21.03 | 9.80-45.15 |
| CPD (baseline diary)^g^ | | −0.12 (0.03) | <.001 | 0.88 | 0.84-0.93 | −0.15 (0.03) | <.001 | 0.86 | 0.82-0.91 |
| Daily NRP^h^ (0=no;  1=yes) | | 1.59 (0.44) | <.001 | 4.90 | 2.05-11.68 | 0.88 (0.51) | .08 | 2.41 | 0.89-6.49 |
| Weekend^i^ (0=no; 1=yes) | | −0.10 (0.11) | .33 | 0.90 | 0.73-1.11 | 0.10 (0.11) | .39 | 1.10 | 0.89-1.37 |
| Group (0=control group; 1=intervention group) | | 1.34 (0.55) | .01 | 3.83 | 1.31-11.24 | 0.09 (0.55) | .87 | 1.10 | 0.37-3.22 |
| Time^j^ | | −0.06 (0.04) | .15 | 0.95 | 0.88-1.02 | −0.05 (0.03) | .13 | 0.95 | 0.89-1.02 |
| Group×time | | 0.04 (0.06) | .50 | 1.04 | 0.93-1.16 | 0.04 (0.05) | .40 | 1.04 | 0.95-1.14 |
| Quit (0=after quit date; 1=before quit date) | | −6.34 (0.71) | <.001 | 0.002 | 0.00-0.01 | −3.95 (0.58) | <.001 | 0.02 | 0.01-0.06 |
| Group×quit | | −0.24 (0.90) | .79 | 0.78 | 0.13-4.59 | −0.74 (0.76) | .34 | 0.48 | 0.11-2.14 |
| Quit×time | | 0.01 (0.08) | .94 | 1.01 | 0.86-1.18 | 0.12 (0.07) | .09 | 1.13 | 0.98-1.30 |
| **Random effects (variance)** | | | | | | | | | |
| Level 2 (interindividual) | | | | | | | | | |
|  | Intercept | 8.00 (1.41) | <.001 |  | 5.59-11.24 | 6.21 (1.21) | <.001 |  | 4.24-9.12 |
|  | Time | 0.07 (0.01) | <.001 |  | 0.05-0.10 | 0.04 (0.01) | <.001 |  | 0.03-0.06 |
|  | Quit | 16.15 (3.23) | <.001 |  | 10.91-23.91 | 15.73 (3.32) | <.001 |  | 10.40-23.78 |
|  | Quit×time | -^k^ |  |  | - | 0.28 (0.08) | <.001 |  | 0.16-0.48 |
| Level 1 (intraindividual)^l^ | | | | | | | | | |
|  | Residual | 0.37 (0.01) | <.001 |  | 0.35-0.40 | 0.43 (0.01) | <.001 |  | 0.40-0.46 |
|  | Autocorrelation | 0.10 (0.02) | <.001 |  | 0.05-0.14 | 0.07 (0.03) | .004 |  | 0.02-0.12 |

^a^GLMM: generalized linear mixed model.

^b^GLMM A1 (logistic regression): n=150 persons with a maximum of 28 days, n=3003/4536 possible diary entries.

^c^GLMM A2 (logistic regression): n=142 persons with a maximum of 28 days, n=2768/4536 possible diary entries.

^d^b=unstandardized regression coefficients.

^e^OR=odds ratio.

^f^Intercept=level of the outcome for the control group at quit date (day 8).

^g^The grand-mean-centered cigarettes per day (CPD) during the *baseline diary* phase as covariate.

^h^NRP: nicotine replacement products; daily usage of nicotine replacement products (0 = no; 1 = yes) as covariate.

^i^Weekend: weekday versus weekend days (0 = no weekend; 1 = weekend) as covariate.

^j^Linear time trend centered on the quit date (day 8=0).

^k^Due to nonconvergence, no random effects for *group×quit* in all models and for *quit×time* in GLMM A1 could be estimated.

^l^Level-1 random effects indicated that there was considerable variation within individuals (ie, residuals from the level-1 fitted values) and significant autocorrelation in the level-1 residuals (ie, that adjacent days were more similar than days further apart).

**Table S5.** Generalized linear mixed model testing effects of the intervention group versus the control group on smoked cigarettes per day (generalized linear mixed model A3) with covariates daily nicotine replacement products and weekend.

|  | | GLMM^a^ A3^b^: CPD^c^ (n=150) | | | |
| --- | --- | --- | --- | --- | --- |
|  | | b^d^ (SE) | *P* value | RR^e^ | 95% CI |
| **Fixed effects** | | | | | |
| Intercept^f^ | | −0.02 (0.16) | .883 | 0.98 | 0.72-1.33 |
| CPD (*baseline diary*)^g^ | | 0.09 (0.01) | <.001 | 1.09 | 1.07-1.12 |
| Daily NRP^h^ (0=no; 1=yes) | | −1.29 (0.25) | <.001 | 0.27 | 0.17-0.45 |
| Weekend^i^ (0=no; 1=yes) | | −0.06 (0.06) | .352 | 0.94 | 0.84-1.07 |
| Group (0=control group; 1=intervention group) | | −0.73 (0.23) | .001 | 0.48 | 0.31-0.75 |
| Time^j^ | | 0.02 (0.01) | .011 | 1.03 | 1.01-1.04 |
| Group×time | | −0.001 (0.01) | .957 | 0.99 | 0.97-1.03 |
| Quit (0=after quit date; 1=before quit date) | | 2.43 (0.17) | <.001 | 11.38 | 8.16-15.86 |
| Group×quit | | 0.68 (0.23) | .003 | 1.97 | 1.26-3.08 |
| Quit×time | | −0.04 (0.03) | .117 | 0.96 | 0.91-1.01 |
| **Random effects (variance)** | | | | | |
| Level 2 (interindividual) | | | | | |
|  | Intercept | 0.93 (0.13) | <.001 |  | 0.70-1.23 |
|  | Time | -^k^ | - |  |  |
|  | Quit | - | - |  |  |
|  | Quit*Time | - | - |  |  |
| Level 1 (intraindividual)^l^ | | | | | |
|  | Residual | 0.78 (0.02) | <.001 |  | 0.73-0.83 |
|  | Autocorrelation | 0.34 (0.02) | <.001 |  | 0.30-0.38 |

^a^GLMM: generalized linear mixed model.

^b^GLMM A3 (negative binomial): n=150 persons with a maximum of 28 days, n=3003/4536 possible diary entries.

^c^CPD: cigarettes per day.

^d^b=unstandardized regression coefficients.

^e^RR=rate ratio.

^f^Intercept=level of the outcome for the control group at quit date (day 8).

^g^The grand-mean-centered cigarettes per day (CPD) during the *baseline diary* phase as covariate.

^h^NRP: nicotine replacement products; daily usage of nicotine replacement products (0 = no; 1 = yes) as covariate.

^i^Weekend: weekday versus weekend days (0 = no weekend; 1 = weekend) as covariate.

^j^Linear time trend centered on the quit date (day 8=0).

^k^Due to non-convergence, only a random intercept in GLMM A3 could be estimated.

^l^Level-1 random effects indicated that there was considerable variation within individuals (ie, residuals from the level-1 fitted values) and significant autocorrelation in the level-1 residuals (ie, that adjacent days were more similar than days further apart).

**Table S6.** Generalized linear mixed models testing effects of the intervention group versus the control group on daily self-reported abstinence and carbon monoxide abstinence (generalized linear mixed models A4 and A5) without drop-outs coded smoking.

|  | | GLMM^a^ A4^b^: daily self-reported abstinence (n=150) | | | | GLMM A5^c^: daily carbon monoxide abstinence (n=142) | | | |
| --- | --- | --- | --- | --- | --- | --- | --- | --- | --- |
|  | | b^d^ (SE) | *P* value | OR^e^ | 95% CI | b (SE) | *P* value | OR | 95% CI |
| **Fixed effects** | | | | | | | | | |
| Intercept^f^ | | 1.27 (0.36) | <.001 | 3.56 | 1.74-7.26 | 3.14 (0.39) | <.001 | 22.98 | 10.78-48.98 |
| CPD (baseline diary)^g^ | | −0.12 (0.03) | <.001 | 0.89 | 0.84-0.94 | −0.15 (0.03) | <.001 | 0.87 | 0.82-0.91 |
| Group (0=control group; 1=intervention group) | | 1.26 (0.54) | .02 | 3.51 | 1.23-10.02 | 0.04 (0.55) | .94 | 1.05 | 0.36-3.04 |
| Time^h^ | | −0.05 (0.04) | .18 | 0.95 | 0.88-1.02 | −0.05 (0.03) | .14 | 0.95 | 0.89-1.02 |
| Group×time | | 0.04 (0.06) | .51 | 1.04 | 0.93-1.16 | 0.04 (0.05) | .41 | 1.04 | 0.95-1.14 |
| Quit (0=after quit date; 1=before quit date) | | −6.41 (0.70) | <.001 | 0.002 | 0.00-0.01 | −3.97 (0.58) | <.001 | 0.02 | 0.01-0.06 |
| Group×quit | | −0.16 (0.89) | .86 | 0.85 | 0.15-4.87 | −0.69 (0.76) | .37 | 0.50 | 0.11-2.24 |
| Quit×time | | −0.01 (0.08) | .93 | 0.99 | 0.85-1.16 | 0.13 (0.07) | .08 | 1.14 | 0.99-1.31 |
| **Random effects (variance)** | | | | | | | | | |
| Level 2 (interindividual) | | | | | | | | | |
|  | Intercept | 7.54 (1.35) | <.001 |  | 5.32-10.7 | 6.19 (1.20) | <.001 |  | 4.22-9.06 |
|  | Time | 0.07 (0.01) | <.001 |  | 0.05-0.10 | 0.04 (0.01) | <.001 |  | 0.03-0.06 |
|  | Quit | 15.86 (3.17) | <.001 |  | 10.71-23.47 | 15.70 (3.32) | <.001 |  | 10.38-23.75 |
|  | Quit×time | -^i^ |  |  | - | 0.28 (0.08) | <.001 |  | 0.16-0.47 |
| Level 1 (intraindividual)^j^ | | | | | | | | | |
|  | Residual | 0.38 (0.01) | <.001 |  | 0.35-0.40 | 0.43 (0.01) | <.001 |  | 0.40-0.45 |
|  | Autocorrelation | 0.10 (0.02) | <.001 |  | 0.05-0.14 | 0.07 (0.03) | .004 |  | 0.02-0.12 |

^a^GLMM: generalized linear mixed model.

^b^GLMM A4 (logistic regression): n=150 persons with a maximum of 28 days, n=3003/4200 possible diary entries.

^c^GLMM A5 (logistic regression): n=142 persons with a maximum of 28 days, n=2768/4200 possible diary entries.

^d^b=unstandardized regression coefficients.

^e^OR=odds ratio.

^f^Intercept=level of the outcome for the control group at quit date (day 8).

^g^The grand-mean-centered cigarettes per day (CPD) during the *baseline diary* phase as covariate.

^h^Linear time trend centered on the quit date (day 8=0).

^i^Due to non-convergence, no random effects for *group×quit* in all models and for *quit×time* in GLMM A4 could be estimated.

^j^Level-1 random effects indicated that there was considerable variation within individuals (ie, residuals from the level-1 fitted values) and significant autocorrelation in the level-1 residuals (ie, that adjacent days were more similar than days further apart).

**Table S7.** Average self-reported daily app use, daily objective app use and daily reported perceived usefulness of the SmokeFree buddy app in the intervention group during the challenge diary phase.

| Variables | *n* | *mean* (*SD*) | *range* |
| --- | --- | --- | --- |
| Self-reported daily app use prequit^a^ | 71^b^ | 1.7 days (1.5) | 0 – 6 |
| Self-reported daily app use postquit | 71 | 5.3 days (4.2) | 0 – 18 |
| Objective app use prequit | 47^c^ | 1.5 minutes (1.35) | 0 – 5.3 |
| Objective app use postquit | 47 | 1.0 minutes (1.0) | 0 – 3.6 |
| Daily perceived usefulness^d^ | 71 | 2.14 (0.82) | 1 – 4.7 |

^a^Daily app use was assessed with the item “Today, I used the SmokeFree buddy app” (0=no; 1=yes).

^b^Available data of 71 participants during the challenge diary phase (10 participants dropped out before the start of the challenge diary).

^c^Objective app use of 47 participants with informed consent.

^d^To assess daily perceived usefulness of the SmokeFree buddy app, every evening participants answered the item “Today I found the Buddy App absolutely useful for my goal to not smoke” on a scale from 1 (today not at all useful) to 6 (today completely useful).
